# Pneumococcal meningitis trends after pneumococcal conjugate vaccine introduction in Colombia: An interrupted time-series analysis

**DOI:** 10.1080/21645515.2018.1425115

**Published:** 2018-02-12

**Authors:** Diana C. Caceres, Eduardo Ortega-Barria, Javier Nieto, Rodrigo DeAntonio

**Affiliations:** a GSK, Bogotá, Colombia; b GSK, Panamá City, Panamá

**Keywords:** Pneumococcal conjugate vaccine, meningitis, Colombia

## Abstract

Colombia introduced mass pneumococcal conjugate vaccination at the end of 2011. Using 2005–2015 surveillance data, we conducted a retrospective interrupted time-series analysis. A significant trend towards reduced monthly was observed in the post-vaccination period (2012–2015) compared with the expected rate, reaching in 2015 a reduction of 90.5% of pneumococcal meningitis. This trend was not observed for control diseases.

*Streptococcus pneumoniae* is a leading cause of bacterial pneumonia, meningitis and sepsis, and has been estimated to cause 11% of all deaths in children aged 1–59 months worldwide.^1^ The World Health Organization recommends pneumococcal conjugate vaccines (PCV) in routine childhood immunization programs in all countries.^2^ In 2006, the Colombian Ministry of Health recommended the use of PCV in high-risk groups (children with birth weight <2,500g, asplenia, sickle cell disease or human immunodeficiency virus, immunocompromised, undergoing transplant or central nervous system surgery, chronic diseases) and departments with lower socioeconomic status, using 7-valent PCV (PCV-7). National vaccine coverage (defined as completion of the recommended PCV schedule) was 16% in 2009, 22% in 2010 and 46% in 2011.^3^ At the end of 2011, PCV-7 vaccination was fully integrated into the Colombian universal mass vaccination (UMV) program using a 2+1 schedule with doses at age 2, 4 and 12 months.^3^ In January 2012, the pneumococcal non-typeable *Haemophilus influenzae* protein D conjugate vaccine replaced PCV-7 in the UMV,^3^ using a 2,4, 12 months of age schedule, without any catch-up. National coverage increased from 16% in 2009 to 46% in 2011 (pre-universal vaccination), and reached 89% in 2014.^3^ The World Health Organization recommends that the impact of vaccination on disease occurrence should be assessed in countries that introduce vaccine programmes.^2^

Public Health Surveillance System was implemented for the Government of Colombia in 1996, and since 2004 the Colombian National Public Health Surveillance System (SIVIGILA) is being in place the National Health Institute, aiming to provide systematic and timely provision of information on the dynamics of events that affect or may affect the health of the Colombian population, in order to guide policies, planning and decision making in public health, monitoring and evaluating the interventions, including those of mandatory report according to international guidelines, and to optimize available resources. The system collects data from all regions in the country for mandatory notifiable diseases including meningitis and pertussis disease. The notification compliance by unit is followed by the National Health Institute and the report of cases is mandatory. Even that is considered there is a sub-register of cases, the reporting process has been stable over time and there are standardized definitions, criteria, diagnosis and notification process through guidelines. Meningitis cases are required to be reported and confirmed by laboratory analysis.^4^ Our objective was to use national disease surveillance data to conduct a retrospective analysis of trends in pneumococcal meningitis in Colombia, before and after the introduction of the PCV program.

Data were obtained from SIVIGILA on the monthly number of laboratory-confirmed cases of pneumococcal meningitis in all age groups for the period 2005–2015. Data on the number of laboratory-confirmed cases of *Haemophilus influenzae* type B (Hib) meningitis and pertussis disease were also obtained from SIVIGILA for all age groups as controls. Pertussis vaccination (included in the diphtheria-tetanus-pertussis vaccine) was introduced in 1977 and Hib vaccination in 1998. Population data were obtained from the National Administrative Department of Statistics^5^ database for the same study period. Monthly number of pneumococcal meningitis cases, and incidence rates estimated using population data from the National Administrative Department of Statistics, were plotted for the period 2005–2015 and compared between the pre-vaccination (2005-2008), PCV-7 in high-risk groups (2009-2011) and the post-UMV periods (2012-2015). An autoregressive integrated moving average (ARIMA) model was developed in November 2016 to assess the impact of the vaccination program by comparing observed incidence rates post- vaccination with the expected incidence rates derived from the model. The ARIMA method is a variation of interrupted time series analysis. A statistical model was fitted to observed time series data using regression, and the fitted model was used to predict expected future trends. The analysis was carried out using STATA and RStudio software.^6^ The observed pneumococcal meningitis incidence data were compared with the expected incidence rates projected by the ARIMA model. These trends were compared with those for the two control diseases, Hib meningitis and pertussis. The interrupted time series analysis estimates cases expected according to the time series behaviour before intervention (contrafactual) and cases observed after the intervention (real number reported to the system). The percentage of reduction is the difference estimated by the model between these 2 values.

A total of 1,056 pneumococcal meningitis cases were reported in Colombia across all age groups in the study period 2005–2015. The number of cases reported each year (2005 to 2015) was 41, 59, 83, 107, 109, 118, 190, 128, 86, 130 and 5, respectively. There was a trend towards increasing incidence until 2012. Since 2012, the trend has been reversed and a reduction in monthly pneumococcal incidence rates was observed in the post-vaccination period (2012–2015) (Fig. 1). The difference was statistically significant (pre-2012, trend 1.28, standard error 0.18; post-2012, trend –3.36, standard error 0.47, p = 0.001). Compared with the expected level if the pre-2012 trends had continued, a reduction of 34.9% in pneumococcal meningitis incidence was observed in the first post-UMV year (2012), followed by reductions of 57.1% in the second year (2013), 75.3% in the third year (2014) and 90.5% in the fourth year (2015). No similar change in the fitted observed trend was observed for the control diseases Hib meningitis or pertussis (Fig. 1).

**Figure 1.**
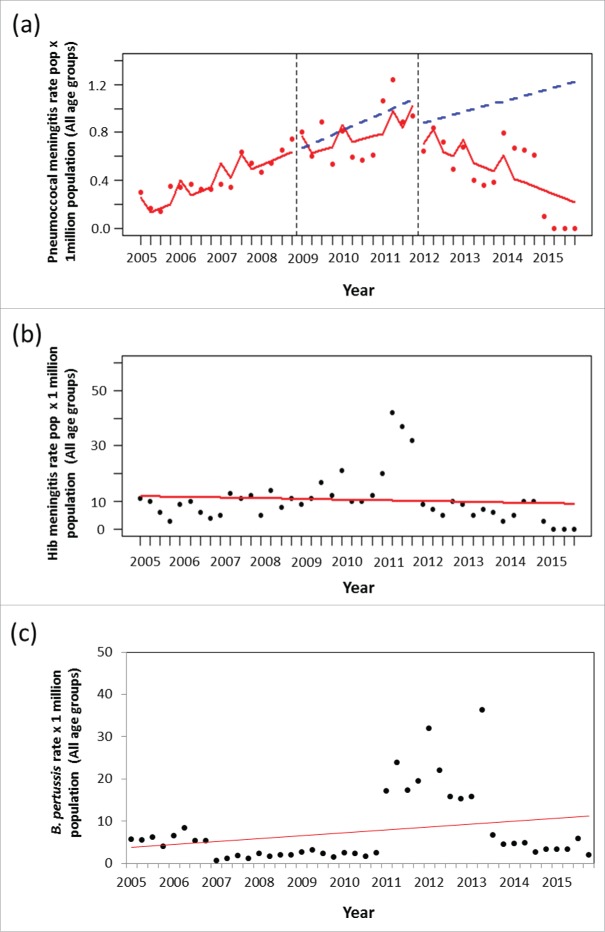
Number of cases of (A) pneumococcal meningitis, (B) *Haemophilus influenzae* type b (Hib) meningitis, (C) pertussis reported to surveillance in Colombia. The vertical dotted lines indicate the introduction of PCV vaccination in high-risk groups and in the universal vaccination program. PCV, pneumococcal conjugate vaccine.

The results of our analysis indicate that the number of pneumococcal meningitis cases in Colombia showed an increasing trend until the year 2012. During 2009–2011 PCV7 coverage rates increased from 16% to 46%. During this period, an apparent non-significant small trend towards reduction during this period (observed vs expected). However, it is not possible to know if it is due to the modest modification obtained with the partial use of PCV7 to be detected by the model (power) or because there is no modification at all with the partial use of the vaccine. After introduction of universal PCV vaccination in 2011, this increasing trend reversed and the number of pneumococcal meningitis cases showed a significant reduction during the period 2012–2015. In 2014, the reduction of 75.3%, was even greater than in 2013. By the fourth year after universal vaccine introduction (2015), the observed number of pneumococcal meningitis cases was reduced compared to the expected number. In the last three quarters of 2015, no cases were reported. The small number of cases observed in 2015 could be influenced by timeliness of reporting, because cases report must be go from municipalities onto the department and national levels; if that were the case, there would be an overestimation of the effect (impact of vaccination). However, the time series show a progressive reduction in the rates of meningitis from 2012 to 2015 and even without considering the last analysed year (2015), the reduction observed after intervention is important (75%). These results support the impact of the pneumococcal vaccine after introduction and are less likely to be attributable to other factors because no similar trends were identified for Hib meningitis or pertussis. A strength of this study is using as comparison data of two other vaccine-preventable diseases with immunization programs that were well established before PCV was introduced, and for which reporting was also mandatory through SIVIGILA. These diseases showed no comparable reduction trend in incidence, thus indicating that the observed decline in pneumococcal meningitis could be linked to PCV introduction and might not be explained only by the report modification or other public health improvements. Even that is expected low incidence of Hib meningitis and pertussis has experienced modifications in the diagnosis during the last years, the aim of these controls was to identify modification in the reporting system which could affect the time series analysis. The selected diseases allow us to meet these objectives. Other diseases could be more appropriate controls for its prevalence or behaviour, however, the mandatory reporting system only includes a limited number of selected diseases. Public health improvements such as changes in healthcare access, improvements in sanitary conditions or changes in surveillance including diagnostics, would also be expected to have an impact on these diseases. As noted in the figure, no changes towards reduction have been observed for these diseases, suggesting that the change in trend observed for pneumococcal meningitis might be attributable to vaccination.

This study demonstrates that analysis of routinely collected mandatory surveillance data on the number of reported cases of infectious diseases is an effective way to monitor trends in disease incidence before and after vaccine introduction. However, it is important to ensure that there are no changes to surveillance methods, case definition, laboratory diagnosis or other variables during the study period that could confound the results. In the present study, the surveillance program in Colombia remained stable throughout the selected study period.^4^ Despite increased awareness of pneumococcal disease, incidence rate was lower compared with previous years.

The initial introduction of PCV-7 for high-risk groups had limited coverage and might have had limited impact on the trend of increasing incidence of pneumococcal meningitis. Coverage increased dramatically after the vaccination program was extended to UMV in 2011, reaching 46% in 2011 and 89% in 2014.^3^ Coverage for diphtheria-tetanus-pertussis and Hib vaccination in 2005–2015 ranged from 85% to 93%, respectively.^7^ Our results are consistent with other reports of the impact of universal PCV vaccination programs in Latin America. In Uruguay, PCV-7 was introduced in 2008 and replaced by PCV-13 in 2010, both using a 2+1 schedule with a catch-up program.^8^ Coverage for the three doses was estimated at 93%.^8^ Analysis of data from 2005–2014 showed a 63.5% reduction in pneumococcal meningitis hospitalizations in children aged 0–14 years in the post-vaccination period compared with pre-vaccination.^8^ In Brazil, pneumococcal non-typeable *Haemophilus influenzae* protein D conjugate vaccine vaccination with a 3+1 schedule was introduced in 2010, and evidence from before-and-after studies indicated decreases of 13–63% in pneumococcal meningitis and 55–77% in pneumococcal meningitis deaths in children aged <3 years.^9^ Another strength of the current analysis is its use of mandatory and unchanged nationwide surveillance data to compare disease incidence pre- and post-vaccination. These data represent a good opportunity to analyse disease incidence and trends after UMV. The regression model projected the number of cases expected from previous trends, and compared this with the observed data. This provides a broad picture of disease occurrence across the whole of Colombia, and can be further updated in the future as more data become available. Our study also has a number of limitations. It is an ecological analysis with no individual data, so it cannot determine whether identified cases of pneumococcal meningitis had received vaccination and is not possible to adjust by other public health intervention which can impact the incidence of pneumococcal disease as a reduction in smokers, taxes for cigarettes, parenteral education, etc. However, the trend changed just after the vaccination started and magnitude in the reduction is so import that could not be explained only by the impact of other public health interventions; even though, is not possible either, determine or dismiss the influence of other public health intervention and its magnitude. The universal PCV vaccination program is targeted to children aged <1 year, but the surveillance data are available only for all age groups combined. Thus, the distribution of cases by age group was not analyzed. There are also limitations around sub-registry and timeliness of reporting in national surveillance systems.^4^ A significant trend to reduction in pneumococcal meningitis rates across all age groups was observed following the introduction of a UMV program in Colombia, reaffirming the benefit of pneumococcal vaccination in the population.

## Abbreviations

ARIMA: autoregressive integrated moving average
Hib: *Haemophilus influenzae* type B
PCV: pneumococcal conjugate vaccine
SIVIGILA: Colombian National Public Health Surveillance System
UMV: universal mass vaccination
